# Leishmanicidal and immunomodulatory activity of *Terminalia catappa* in *Leishmania amazonensisin vitro* infection

**DOI:** 10.1016/j.heliyon.2024.e24622

**Published:** 2024-01-12

**Authors:** Sandra Alves de Araújo, Carla Maria Pereira Silva, Carolina Silva Costa, Clarissa Sousa Costa Ferreira, Helen Silva Ribeiro, Aldilene da Silva Lima, Cláudia Quintino da Rocha, Kátia da Silva Calabrese, Ana Lucia Abreu-Silva, Fernando Almeida-Souza

**Affiliations:** a Rede Nordeste de Biotecnologia, Universidade Federal do Maranhão, São Luís, 65080-805, Brazil; b Universidade Estadual do Maranhão, São Luís, 65055-310, Brazil; c Laboratório de Química dos Produtos Naturais, Universidade Federal do Maranhão, 65080-805, São Luís, MA, Brazil; d Laboratório de Protozoologia, Instituto Oswaldo Cruz, Fiocruz, Rio de Janeiro, 21041-250, Brazil; e Pós-graduação em Ciência Animal, Universidade Estadual do Maranhão, São Luís, 65055-310, Brazil

**Keywords:** *Leishmania amazonensis*, Natural products, Flavonoid, Ellagic acid, Immunomodulation, Treatment

## Abstract

Leishmaniases are infectious-parasitic diseases that impact public health around the world. Antileishmanial drugs presented toxicity and increase in parasitic resistance. Studies with natural products show an alternative to this effect, and several metabolites have demonstrated potential in the treatment of various diseases. *Terminalia catappa* is a plant species with promising pharmaceutical properties. The objective of this work was to evaluate the therapeutic potential of extracts and fractions of *T. catappa* on *Leishmania amazonensis* and investigate the immunomodulatory mechanisms associated with its action. In anti-*Leishmania* assays, the ethyl acetate fraction exhibited activity against promastigotes (IC_50_ 86.07 ± 1.09 μg/mL) and low cytotoxicity (CC_50_ 517.70 ± 1.68 μg/mL). The ethyl acetate fraction also inhibited the intracellular parasite (IC_50_ 25.74 ± 1.08 μg/mL) with a selectivity index of 20.11. Treatment with *T. catappa* ethyl acetate fraction did not alter nitrite production by peritoneal macrophages stimulated with *L. amazonensis,* although there was a decrease in unstimulated macrophages treated at 50 μg/mL (p = 0.0048). The *T. catappa* ethyl acetate fraction at 100 μg/mL increased TNF-α levels (p = 0.0238) and downregulated HO-1 (p = 0.0030) and ferritin (p = 0.0002) gene expression in *L. amazonensis*-stimulated macrophages. Additionally, the total flavonoid and ellagic acid content for ethyl acetate fraction was 13.41 ± 1.86 mg QE/g and 79.25 mg/g, respectively. In conclusion, the *T. catappa* ethyl acetate fraction showed leishmanicidal activity against different forms of *L. amazonensis* and displayed immunomodulatory mechanisms, including TNF-α production and expression of pro and antioxidant genes.

## 1 Introduction

Leishmaniases are neglected tropical diseases of great importance in public health caused by different parasites species of the genus *Leishmania* (family Trypanosomatidae) [1,2]. Worldwide, about 12–15 million people have the disease. In 2021, Brazil led the number of annual cases among countries in South America, reporting about 15,023 cases [3]. The diseases presents different clinical manifestations that directly influence the severity and response to treatment [4,5].

Currently, the treatment of leishmaniasis is based on limited number of drugs. Pentavalent antimonials, amphotericin B, miltefosine, paromomycin and pentamidine are the only treatment options for the disease [5]. These drugs have been in use for decades, contributing to the development of parasite resistance. Besides toxicity and high costs of these drugs, they are challenging to administer to the patients [6,7]. To date, there is no vaccine and no suitable medicine available to prevent the disease. Thus, the search for more effective treatments without side effects has become ever greater. Based on this, new possibilities for the control and treatment of leishmaniasis have emerged.

The therapeutic potential of medicinal plant extracts and their applicability in the treatment of various diseases demonstrate the importance of the discovery of new bioactive molecules [[8], [9], [10]]. Additionally, natural plant-derived compounds possess immunomodulatory properties against antiparasitic diseases, including leishmaniasis [11]. *Terminalia* is a botanical genus belonging to the Combretaceae family with about 250 described species [12,13]. *Terminalia catappa* species popularly known as “amendoeira” is the most common species of the genus in Brazil, plentifully found in the Northeast region of the country [14].

**Fig. 1:**
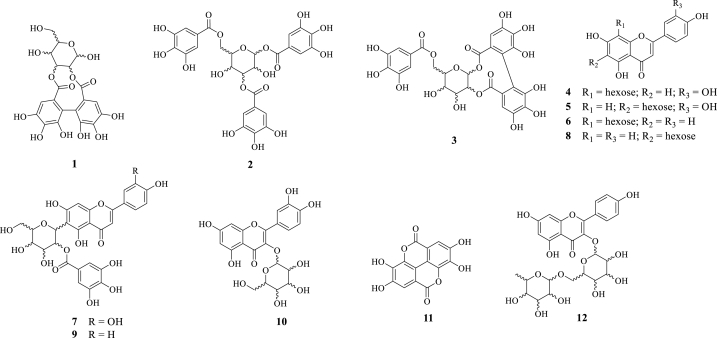
Structures of chemical compounds identified in the ethyl acetate fraction of *Terminalia catappa*. 1: Hexahydroxydiphenyldigalloylglucose acid; 2: Trigaloyl-hexoside 3: Galoyl-HHDP-hexoside 4: Luteolin 8-*C*-hexoside; 5: Luteolin 6-*C*-hexoside; 6: Apigenin 8-*C*-hexoside; 7: Luteolin-6*-C*-(2″-galloyl)-hexoside 8: Apigenin 6-*C*-hexoside 9: Apigenin 6*-C*-(2″-galloyl)-hexoside; 10: Quercetin 3-*O*-hexoside 11: Ellagic acid; 12: Kaempferol 3-*O*-(6″-deoxyhexosyl) -hexoside.

Phytochemically, *T. catappa* species is known to be a source of polyphenolic compounds [15,16]. Flavonoids are the most abundant compounds in *Terminalia* species [16]. Our recent study demonstrated a variety of flavonoids and polyphenols identified in the ethyl acetate fraction of *T. catappa*, such as apigenin, luteolin, quercetin, kaempferol and ellagic acid (Fig. 1) [17].

Different biological activities have been described for *T. catappa*, including antihelmintic [15,18]; anti-diabetic [19], antifungal [20], anti-inflammatory [21], and anticancer [22]. Our previous study with *T. catappa* described the *in vitro* antioxidant and antitrypanosomal activities against *Trypanosoma cruzi*, demonstrating that the extract and fractions showed action against all forms of the parasite and no toxicity to host cells [17]. In addition, we observed ultrastructural damage in epimastigote forms of *T. cruzi* treated with the ethyl acetate fraction, showing the plant potential against protozoan parasites. Based on the similarity between the parasites of the Trypanosomatidae family and the absence of studies demonstrating the leishmanicidal activity of *T. catappa*, this study aimed to investigate the potential of the extract and fractions of *T. catappa* and to identify the effect of the treatment on the immunomodulatory responses against *Leishmania amazonensis* infection *in vitro*.

## 2 Materials and method

### 2.1 Plant material

The details regarding the collection, extraction, fractioning, and phytochemical analysis of *T. catappa* are described in Ref. [17]. In summary, the initial step involved drying and grinding of fresh leaves. The extraction process utilized 70 % ethanolic alcohol by percolation. The resulting hydroethanolic extract was separated into fractions using solvents with varying polarities (hexane, ethyl acetate, and water/methanol) (Dinâmica, Indaiatuba, BRA). Solvents were removed from both the crude extract and fractions using a rotary evaporator under controlled temperature, followed by lyophilization. Samples of the extract and fractions were diluted in dimethyl sulfoxide (DMSO; Sigma, St. Louis, MO, USA) at a stock concentration of 100 mg/mL and stored at −20 °C until it is required for the biological assay. DMSO final concentration did not exceed 1 % in all biological assays.

### 2.2 Parasites

*Leishmania amazonensis* H21 (MHOM/BR/76/MA-76) promastigote forms were cultured at 26 °C in Schneider's Insect medium (Sigma, St. Louis, MO, USA), supplemented with 10 % fetal bovine serum (FBS) (Gibco, Gaithersburg, MD, USA), penicillin (100 U/mL) (Gibco, Gaithersburg, MD, USA) and streptomycin (100 μg/mL) (Sigma, St. Louis, MO, EUA).

### 2.3 Activity against promastigote forms

*L. amazonensis* promastigote forms (10^6^ parasites/mL) were plated in 96-well plates and treated with hydroethanolic extract, hexane, ethyl acetate and water-methanol fractions or ellagic acid at different concentrations (500–15.62 μg/mL) diluted in Schneider's medium, with a final volume of 100 μL per well, and incubated at 26 °C for 72 h. Wells with no parasites, and wells with only parasites plus 1 % DMSO, were used as a blank and negative control, respectively. Amphotericin B (Sigma, St. Louis, MO, USA; 1.25 to 0.0195 μg/mL) was used as the reference drug. After the incubation time, the viability of the promastigote forms was verified by counting in a Neubauer chamber. The experiment was carried out in triplicate. The results were expressed by the 50 % growth inhibitory concentration of the parasite (IC_50_) in relation to the untreated parasite following the formula: IC_50_ = (sample counting)/(control counting) × 100.

### 2.4 Animals

Male BALB/c mice 6 weeks-old were acquired from the Institute of Science and Technology in Biomodels of Oswaldo Cruz Institute. All mice were maintained under controlled temperatures, with a light cycle of 12 h light/dark, at 25 ± 2 °C, and with relative humidity ranging between 55 and 65 %. The animals received food and water *ad libitum* during the experiments as approved by the Ethics Committee on Animal Care and Utilization of the Oswaldo Cruz Institute, CEUA-IOC license n° L53/2016-A3.

### 2.5 Peritoneal Macrophage obtaining and cell culture

Macrophages were obtained by peritoneal lavage of BALB/c mice previously elicited with 3 mL of 3 % sodium thioglycolate (Sigma, St. Louis, MO, USA). After 72 h of stimulation, the animals were euthanized with 10 % ketamine (Syntec, Barueri, BRA) and 2 % xylazine (Syntec, Barueri, BRA) at high doses. Macrophages were harvested from the peritoneal cavity with 10 mL of cold sterile PBS solution (pH 7.2), centrifuged at 210×*g* at 4 °C for 5 min and resuspended in RPMI 1640 medium (Sigma, St. Louis, MO, USA), supplemented with 10 % FBS, penicillin (100 U/mL) and streptomycin (100 μg/mL), at 37 °C and 5 % CO_2_.

### 2.6 Cytotoxicity assay

BALB/c peritoneal macrophages were incubated in a 96-well plate (5 x 10^5^ cells/mL) for cell adhesion overnight (37 °C, 5 % CO_2_). In triplicate, cells were treated with different concentrations of hydroethanolic extract, hexane, ethyl acetate and water-methanol fractions (1000–31.25 μg/mL) and incubated for 24 h. Amphotericin B was used as the reference drug (5–0.3125 μg/mL). Wells without cells were used as blank and wells with cells plus 1 % DMSO were used as control. Afterwards, cytotoxicity was assessed by adding 10 μL MTT (5 mg/mL) for 2 h [23]. The supernatant was discarded and the formazan crystals were dissolved by addition of 50 μL DMSO. Finally, absorbance at 570 nm was measured using a Biochrom EZ Read 400 plate reader (Biochrom, Cambridge, UK). Data were normalized according to the following formula: % survival = (Abs. sample-Abs. blank)/(Abs. control-Abs. blank) x 100. The cytotoxic concentration for 50 % of the peritoneal macrophages (CC_50_) was then calculated. The selectivity index (SI) was calculated from the ratio of CC_50_ for murine peritoneal macrophages by the IC_50_ for the *L. amazonensis* promastigote forms, according to the equation: SI_pro_ = CC_50_/IC_50._

### 2.7 Activity against intracellular amastigote forms

BALB/c peritoneal macrophages were cultured in 24-well plates (5 x 10^5^ cells/well) containing round coverslips, and incubated overnight (37 °C, 5 % CO_2_). The cells were infected with *L. amazonensis* metacyclic promastigote forms (10:1 ratio, parasites/cell) for 1 h at 35 °C, 5 % CO_2_. Non-internalized parasites were removed by washing. In quadruplicate, infected cells were treated with *T. catappa* ethyl acetate fraction at different concentrations (500–15.62 μg/mL) and the plates were incubated under the same conditions at 37 °C for 24 h. Amphotericin B (5–0.3125 μg/mL) was used as reference drug and infected cells treated with 1 % DMSO were used as controls. After infection and treatment, coverslips were fixed in Bouin's solution (Sigma, St. Louis, MO, USA) and stained with Giemsa solution (Sigma, St. Louis, MO, USA) to evaluate infection and treatment efficiency. Infection rate, mean number of amastigotes/cell and total number of amastigotes/200 cells were calculated [24]. The IC_50_ was calculated from the intracellular amastigote count in 200 cells in comparison with the untreated control. The selectivity index (SI) was calculated from the ratio of CC_50_ for murine macrophages with the IC_50_ for the *L. amazonensis* amastigote forms, according to the equation: SI_ama_ = CC_50_/IC_50_.

### 2.8 Stimulation of peritoneal macrophages with L. amazonensis and T. catappa

In 24-well plate, BALB/c peritoneal macrophages (2 x 10^6^/well) were stimulated by *L. amazonensis* metacyclic promastigote forms (10:1 ratio, parasites/cell) for 1 h at 35 °C in 5 % CO_2_. After, *L. amazonensis*-stimulated and non-stimulated cells were treated with *T. catappa* ethyl acetate fraction (100, 50 and 25 μg/mL) for 72 h at 37 °C. Macrophages stimulated and non-stimulated with *L. amazonensis* were used as reference. Cells stimulated with lipopolysaccharide (LPS, Sigma, St. Louis, MO, USA) at 10 μg/mL was used as positive control. The supernatant was collected to measure nitrite and cytokine production, and RNA was extracted from the adherent cells to evaluated pro- and antioxidant gene expression.

### 2.9 Nitrite and Cytokine quantification

Nitrite levels in the supernatant were determined by Griess reagent (N-(1-naphthyl) ethylenediamine dihydrochloride 0.1 % solution and sulfanilamide 1 % in 2.5 % H_3_PO_4_ solution). The amount of NaNO_2_ (Sigma, St. Louis, MO, USA) was calculated by comparison with a standard curve (100–0.39 μM) measured at 570 nm. Cytokines quantification of TNF-α, IFN-γ, IL-10, IL-4, IL-1β using BD OptEIA™ kit (Becton, Dickinson, Franklin Lakes, NJ, USA), and IL-12 p70 using DuoSet® ELISA kit (R&D System, Mineápolis, MN, USA) was performed following the manufacturer's specifications.

### 2.10 Gene expression by RT-PCR

Total RNA from *L. amazonensis* stimulated and non-stimulated macrophages that were treated or untreated with *T. catappa* was extracted using TRIzol reagent (Invitrogen, Carlsbad, CA, USA) following the manufacturer's instructions. RNA concentrations were determined by spectrophotometry NanoDrop One (Applied Biosystems, Waltham, MA, USA). First strand cDNA was synthesized with 300 ng of total RNA using iScript™ cDNA Synthesis Kit (Bio-Rad, Hercules, CA, USA) according to the protocol provided by manufacturer. The cDNA was quantified and diluted to the concentration of 50 ng/μL. The reaction was carried out with 2 μL of cDNA, 0.5 μL of each primer at 100 nM, 2 μL of ultra-pure H_2_O and 5 μL of Go-Taq qPCR master mix (Promega, Madison, WI, USA), with the final volume of 10 μL per well. The sequences of the specific primers targeting mouse genes were: nuclear factor, erythroid derived 2-like 2, (Nrf2) (forward 5′TCACACGAGATGAGCTTAGGGCAA3′, reverse 5′TACAGTTCTGGGCGGCGACTTTAT3′), heme oxygenase-1 (HO-1) (forward 5′CCCAAAACTGGCCTGTAAAA 3′, reverse 5′CGTGGTCAGTCAACA TGGAT3′), L-ferritin (ferritin) (forward 5′TTCCAGGATGTGCAGAAGCC3′, reverse 5′AAGAGGGCCTGATTCAGGTTC3′) [25] and ribosomal protein, large, P0 (RPLP0) (foward 5′GCCAGCTCAGAACACTGGTCTA3′, reverse 5′ATGCCCAAAGCCTGGAAGA3′) [26]. The qPCR was performed using QuantStudio 3 equipment (Applied Biosystems, Waltham, MA, USA). Reactions were conducted using a three-step real-time PCR program as follow: hold stage at 95 °C for 2 min, PCR stage at 40 cycles of 95 °C for 15 s and 60 °C for 1 min, and melt curve stage with 95 °C for 1 min, 60 °C for 1 min, and 95 °C for 15 s with temperature raising at 0.1 °C/s. The level of gene expression was calculated by the relative quantification 2^−ΔΔCt^ method, using the mouse RPLP0 gene as endogenous control.

### 2.11 Analysis of total flavonoid content

The total flavonoid content of the *T. catappa* was performed using the 2 % aluminum chloride (AlCl_3_) (Sigma, St. Louis, MO, USA). Initially, a standard curve was constructed using quercetin (Sigma, St. Louis, MO, USA) at different concentrations (1.000–50 μg/mL). In 96-well plate, 100 μL extract and fractions solubilized in methanol (1.000 μg/mL) was mixed with 100 μL of 2 % AlCl_3_ solution and incubated at room temperature in the dark for 30 min. After this period, the absorbance value was measured at 420 nm using a spectrophotometer. All tested solutions were made in triplicate. The amount of flavonoids was expressed in milligrams of quercetin equivalent per gram of extract (mg QE/g).

### 2.12 Quantification of ellagic acid

Initially, stock solutions of the hydroalcoholic extract and ethyl acetate fraction of *T. catappa* were prepared at 5.000 mg/mL in methanol: water (95:5, v/v). Subsequently, they were eluted in solid phase using a Phenomenex Strata C18 cartridge, collected in volumetric flasks, and filtered in a 0.22 μm Millex filter. In triplicate, 10 μL aliquots of the solutions were injected into the HPLC-UV to obtain the peak corresponding to ellagic acid (Sigma, St. Louis, MO, USA). The Luna C18 column (25 cm × 4.6 mm x 5 μm) (Phenomenex, Torrance, CA, USA) with mobile phase gradient composed of water (A): methanol (B) containing 0.01 % formic acid (Sigma, St. Louis, MO, USA) (flow of 1 mL/min; monitoring at 254 nm) was used to separate the compounds. The standard's analytical curve demonstrated linearity within the studied concentration range, with a correlation (R^2^) of 0.9806 (Supplementary material – S1). To determine the quantity of ellagic acid present, we calculated it in milligrams per gram of dry extract and/or fractions (mg/g).

### 2.13 Statistical analysis

The data were expressed by mean ± S.D. and analyzed statistically by Mann-Whitney Test. The analyses were performed with the software GraphPad Prism 8.00 (GraphPad Software Inc., San Diego, CA, USA) and differences were considered significant when p < 0.05.

## 3 Results

### 3.1 Leishmanicidal activity and cytotoxicity

**Fig. 2:**
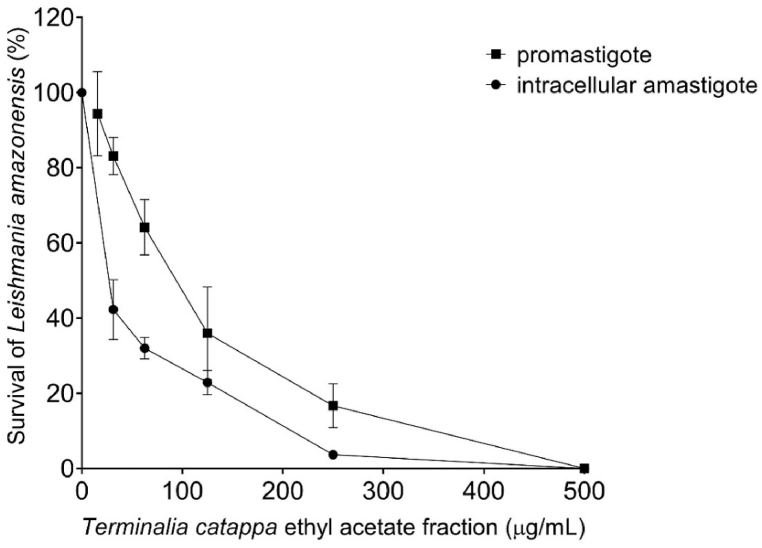
Effects of *Terminalia catappa* ethyl acetate fraction on survival of *Leishmania amazonensis* promastigotes and intracellular amastigotes forms. Data represents the mean ± standard deviation of the experiment realized in triplicate.

The IC_50_ values of leishmanicidal activity against promastigotes and intracellular amastigotes, in addition to cytotoxicity to peritoneal macrophages and SI, are shown in Table 1. *T. catappa* extract and fractions showed different effects on *L. amazonensis* promastigotes forms. The hydroethanolic extract and water-methanol fraction did not demonstrate leishmanicidal activity at the analyzed concentrations. The ethyl acetate fraction was more effective against promastigotes than all the other material evaluated, displaying IC_50_ of 86.07 ± 1.09 μg/mL. Cytotoxicity evaluation showed that the hexane fraction was the most cytotoxic to peritoneal macrophages (IC_50_ of 87.56 ± 1.24 μg/mL), followed by ethyl acetate fraction and ellagic acid. The hydroethanolic extract and water-methanol fraction were not toxic to peritoneal macrophages. In addition, it was observed that the ethyl acetate fraction exhibited a higher selectivity index for promastigotes than hexanic fraction. Thus, the activity against *L. amazonensis* intracellular amastigotes was evaluated only with ethyl acetate fraction, and it was 3.3-fold more effective against intracellular amastigotes than against promastigotes. Additionally, ethyl acetate fraction produced a concentration-dependent reduction in the percentage of survival of both parasite forms, with survival reduction by 100 % at the highest test concentration (Fig. 2). Amphotericin B showed leishmanicidal activity, cytotoxicity and selectivity index as expected.

**Table 1:** Cytotoxic effect, leishmanicidal activity and selectivity index of the extract and fractions of *Terminalia catappa*.

Compounds	Peritoneal macrophage	*Leishmania amazonensis*			
	CC_50_ (μg/mL)	Promastigotes IC_50_ (μg/mL)	SI_pro_	Intracellular amastigotes IC_50_ (μg/mL)	SI_ama_
Hydroethanolic extract	>1000	>500	nd	nd	nd
Hexanic fraction	87.56 ± 1.24	459.20 ± 0.38	0.19	nd	nd
Ethyl acetate fraction	517.70 ± 1.68	86.07 ± 1.09	6.01	25.74 ± 1.08	20.11
Water-methanol fraction	>1000	>500	nd	nd	nd
Ellagic acid	312.20 ± 1.25	210.90 ± 1.66	1.48	nd	nd
Amphotericin B	3.75 ± 1.25	0.04 ± 0.09	93.75	0.10 ± 0.03	37.50

Data represents the mean ± standard deviation of the experiment realized in triplicate. CC_50_: cytotoxic concentration of 50 % cells; IC_50_: inhibitory concentration of 50 % parasites; SI_pro_: selective index for promastigote forms; SI_ama_: selective index for intracellular amastigote forms; nd: not determined.

**Fig. 3:**
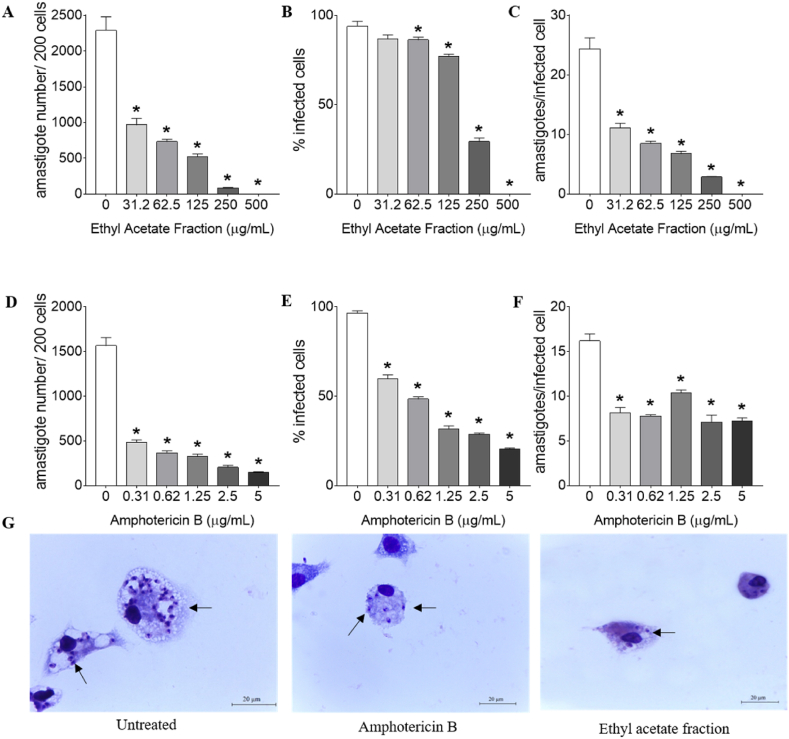
Effect of *Terminalia catappa* ethyl acetate fraction on BALB/c peritoneal macrophages infected with *Leishmania amazonensis*. (A–F) Parameters of infection after 24h of *T. catappa* ethyl acetate fraction or amphotericin B treatment. Data represents mean ± standard deviation of experiment realized in triplicate. *p < 0.05 when compared with untreated infected cells by Mann-Whitney Test. (G) Light microscopy representative of two experiments of untreated infected cells and those treated with amphotericin B (2.5 μg/mL) and ethyl acetate fraction (125 μg/mL). Black arrows point intracellular amastigotes inside macrophages. Giemsa, 100 × objective.

The parameters of *L. amazonensis* infection were calculated in relation to the untreated control cells after 24h of treatment (Fig. 3). The ethyl acetate fraction inhibited the number of parasites in 200 macrophages at concentrations 31.2, 62.5, 125, 250 and 500 μg/mL (Fig. 3A), reduced the number of parasites per infected cell at 31.2, 62.5, 125, 250 and 500 μg/mL concentrations (Fig. 3C), and decreased the percentage of infectivity of macrophages at 62.5, 125, 250 and 500 μg/mL concentrations (Fig. 3B). In infected cells treated with amphotericin B (0.31, 0.625, 1.25, 2.5 and 5 μg/mL), a significant reduction was observed in all parameters of infection (Fig. 3D–F). Fig. 3G shows representative images of BALB/c peritoneal macrophages infected with *L. amazonensis* and treated with *T. catappa* ethyl acetate fraction and amphotericin B.

### 3.2 Quantification of nitrite and cytokine levels

**Fig. 4:**
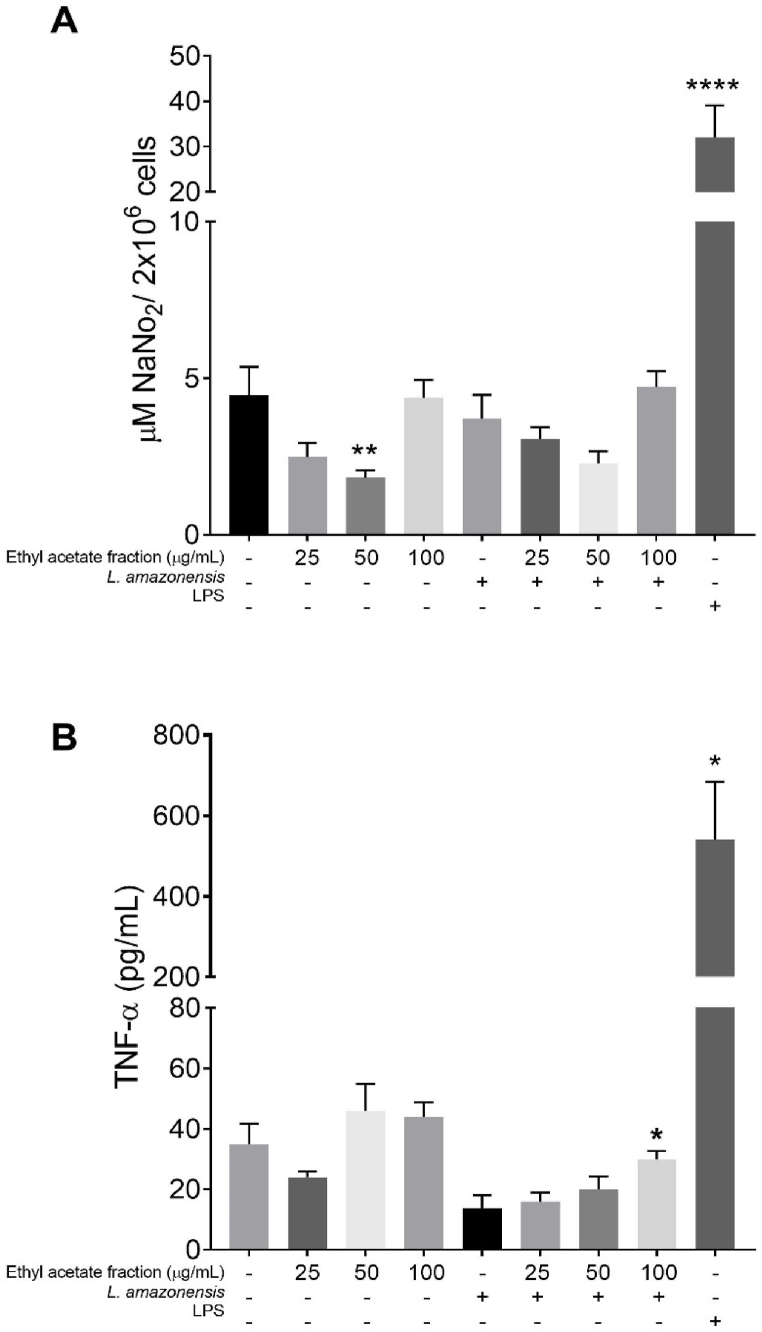
Nitrite (A) and TNF-α (B) quantification in the supernatant of BALB/c peritoneal macrophages treated or not with *Terminalia catappa* ethyl acetate fraction and stimulated or not with *Leishmania amazonensis* or LPS. Data represents mean ± standard deviation of experiment realized in quintuplicate. *p < 0.05; **p < 0.01; ****p < 0.0001 when compared with untreated and unstimulated macrophages by Mann-Whitney test.

The effect of *T. catappa* ethyl acetate fraction on the levels of NaNO_2_ and cytokine was evaluated in the supernatants of BALB/c peritoneal macrophages (Fig. 4). Macrophages produced low levels of nitrite when treated with different concentrations of the fraction. There was a decrease in NaNO_2_ production after 72 h of treatment with the ethyl acetate fraction at 50 μg/mL (1.81 ± 1.40 μM NaNO_2_, p = 0.0048) when compared to untreated cells (4.46 ± 3.01 μM NaNO_2_). However, cells stimulated with *L. amazonensis* and treated with ethyl acetate fraction did not change nitrite levels, although there was a slight decrease at 50 μg/mL (2.27 ± 0.77 μM NaNO_2_) compared to stimulated and non-treated cells (3.72 ± 2.25 μM NaNO_2_). As expected, macrophages stimulated with LPS produced high levels of nitrite (32.01 ± 27.46 μM NaNO_2_, p = 0.0001) (Fig. 4A). The TNF-α quantification is showed in Fig. 4B. After 72 h, *L. amazonensis*-stimulated cells and treated with the ethyl acetate fraction induced an increase in TNF-α levels at 100 μg/mL (30 ± 6.12 pg/mL; p = 0.0238) compared to stimulated and untreated group (13.75 ± 8.53 pg/mL). The other cytokines not exhibited significant alteration after *T. catappa* treatment (Supplementary material - S2). In addition, macrophages stimulated with LPS exhibited increased levels of TNF-α (541 ± 145.05 pg/mL; p = 0.0159).

### 3.3 Nrf2, HO-1 and ferritin expression analysis

**Fig. 5:**
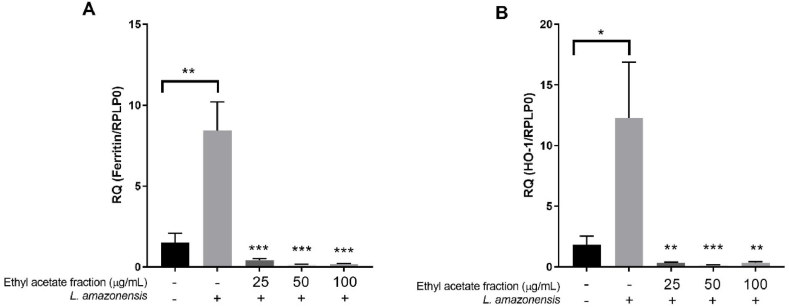
Relative quantification of pro- and antioxidant genes in BALB/c peritoneal macrophages stimulated with *Leishmania amazonensis* and treated with *Terminalia catappa*. RT-qPCR analyses were performed to quantify the expression of ferritin (A), HO-1 (B) mRNA. Expression was estimated by 2^−ΔΔCT^ method, using RPLP0 as reference gene. The bars represent the mean ± standard deviation of experiment performed in triplicate. *p < 0.05, **p < 0.01 and ***p < 0.001 when compared with *L. amazonensis*-stimulated cells or between groups indicated by bracket after statistical evaluation by Mann-Whitney test. HO-1 - hemeoxygenase 1; RPLP0: ribosomal protein, large, P0.

The expression of Nrf2, HO-1 and ferritin mRNA were investigated in cells stimulated with *L. amazonensis* and treated with *T. catappa* (Fig. 5). Ferritin and HO-1 expression were significantly upregulated in the group of cells stimulated with parasites when compared to the non-stimulated group (p = 0.0013 and p = 0.0426, respectively), as observed in Fig. 5A e 5B. The expression of ferritin mRNA in cells treated with the ethyl acetate fraction was significantly reduced at 25, 50, and 100 μg/mL (p = 0.0002) when compared to the stimulated cells (Fig. 5A). HO-1 expression was significantly downregulated in cells treated with 25, 50 and 100 μg/mL of ethyl acetate fraction (p = 0.0017; p = 0.0007 and p = 0.0030, respectively) as shown in Fig. 5B. The treatment with *T. catappa* ethyl acetate fraction with the concentrations evaluated did not alter the expression of Nrf2 (Supplementary material - S3).

### 3.4 Chemical characterization, flavonoid and ellagic acid quantification

After extraction, fractionation and lyophilization, *T. catappa* material originated hydroethanolic extract and hexane, ethyl acetate and water-methanol fractions. The total flavonoid content and ellagic acid of *T. catappa* samples was determined (Table 2). The highest content of flavonoids was observed in the hexane fraction, followed by the water-methanol and ethyl acetate fractions. The lowest flavonoid content was found in the hydroethanolic extract. Additionally, the content of ellagic acid was obtained from the hydroethanolic extract and ethyl acetate fraction, as shown in Table 2.

**Table 2:** Content of total flavonoids and ellagic acid of the extract and fractions obtained from *Terminalia catappa* leaves.

Compounds	Total flavonoids (mg QE/g)	Ellagic acid (mg/g)
Hydroethanolic extract	7.52 ± 1.49	28.29 ± 0.05
Hexane fraction	65.31 ± 12.26	nd
Ethyl acetate fraction	13.41 ± 1.86	79.25 ± 0.59
Water-methanol fraction	14.86 ± 1.38	nd

mg QE/g: milligram of quercetin equivalent per gram of extract; mg/g: milligram per gram of dry extract. Values are expressed as mean ± SD of triplicates; nd: not determined.

## 4 Discussion

Plants of the genus *Terminalia* have several promising biological properties, however the mechanisms involved in their bioactivity are unknown [27,28]. In this study, the leishmanicidal action of extract and fractions of *T. catappa* against *L. amazonensis* and its immunomodulatory activity were investigated.

The biological activities of plant extracts are mainly associated with the presence of constituents of the secondary metabolism of the plant. These phytochemicals are extracted using different organic solvents, including ethanol, ethyl acetate, n-butanol and methanol [29,30]. In the genus *Terminalia*, phenolic compounds are the predominant phytochemical substances [11,12], and flavonoids constitute the major group of phenolics compounds [31,32].

In our previous study, total flavonoids content in the extract and fractions of *T. catappa* was verified and confirmed by HPLC-UV-ESI/MS analysis of the ethyl acetate fraction, identifying a rich flavonoids content with compounds such as luteolin, apigenin, quercetin and kaempferol [17]. Studies conducted by Terças et al. [33] verified the presence of flavonoid C-glycosides, tannins and gallic acid in the N-butanol fraction of *T. catappa* leaves collected in São José de Ribamar, Maranhão, Brazil. In addition, ellagic acid, a polyphenolic compound, was also identified in the *T. catappa* ethyl acetate fraction. Quantitative analysis showed that ellagic acid represented 79.25 mg/g of dry extract in the ethyl acetate fraction. This compound it has been mentioned in other species of *Terminalia* genus [[34], [35], [36]].

The leishmanicidal potential of phenolic compounds is well known and their activity has been attributed to their ability to induce cell death through apoptosis, inhibition of cell replication and induction of mitochondrial damage [37,38]. Flavonoids act increasing reactive oxygen species levels, causing mitochondrial damage to *Leishmania* parasites, intense cytoplasm vacuolization and inducing cell cycle blockage and autophagy [39,40].

During the preliminary screening for activity against the promastigotes forms, the hydroethanolic extract and the water-methanol fraction of *T. catappa* did not exhibit activity against *L. amazonensis*, while the ethyl acetate fraction showed greater antiparasitic activity and less cytotoxic effect compared to the hexanic fraction. In addition, the selectivity index (SI) found for the ethyl acetate fraction indicates greater toxicity for parasites than for macrophages. According to Nwaka and Hudson [41], the SI acceptable for new drug candidates should be close or greater than 20. Therefore, SI results are essential in the search for effective natural products for the treatment of leishmaniasis. Based on that, the *T. catappa* ethyl acetate fraction was evaluated for its anti-*Leishmania* activity.

In the literature, there are no reports on the leishmanicidal activity of the *T. catappa* species. Fungi and protozoa have a common characteristic, ergosterol [42]. Ergosterol is a sterol abundantly found in the cell membranes of organisms whose main function is to regulate fluidity and to contribute to the organization of membrane domains [43]. In *Leishmania*, ergosterol helps the parasites to survive within the aggressive environment of the host macrophage, leading to a successful infection [44,45]. Evidence indicates that amphotericin B, an antifungal drug used in the treatment of leishmaniasis, selectively binds to ergosterol, leading to disruption of the osmotic integrity of the membrane, causing disruption of cell integrity [46]. Compounds that inhibit ergosterol biosynthesis become promising for the treatment of leishmaniasis. The antifungal potential of *T. catappa* reported by other studies [20,33] allows us to correlate our results. Thus, *T. catappa* ethyl acetate fraction may act directly on the cell membrane of *L. amazonensis* promastigotes, causing the destruction of the parasite. Further studies should evaluate this or other mechanisms that may be involved in its action against *Leishmania* parasites.

The different bioactivity of plant extracts is related to the complexity of phytochemical compounds and the combination between them [47]. For example, synergism occurs when the combined effect of compounds is greater than the sum of their individual effects [48]. In this study, the ellagic acid alone did not exhibit good activity against the species *L. amazonensis*, although other studies have observed the antileishmanial potential of ellagic acid against *L. donovani* and *L. major* [49,50]. Thus, we can indicate that synergistic effects or other compound may be responsible for the leishmanicidal action of the ethyl acetate fraction of *T. catappa* against different forms *L. amazonensis*.

Studies with extracellular forms are important in the screening of potential leishmanicidal drugs, however, tests with intracellular forms are extremely necessary as it is the most appropriate way to relate the *in vitro* activity of a compound with a possible *in vivo* efficacy [51]. In our study, *T. catappa* ethyl acetate fraction was 3.3-fold more effective against intracellular amastigotes than against promastigotes, leading us to infer that the compounds acted directly on the parasite, crossing the cell membrane of the macrophage, and indirectly by activating macrophage immunomodulatory mechanisms, such as increasing nitric oxide (NO) and cytokine production and/or activating antioxidant responses.

The NO is one of the most important molecules in the control of *Leishmania* parasites [50,52]. The inhibition of intracellular amastigotes is directly related to NO production by macrophages [52], however, in this study, treatment with *T. catappa* ethyl acetate fraction did not increase NO production. This reveal that NO should not be characterized as the main mechanism involved in leishmanicidal activity against intracellular amastigote of *T. catappa* ethyl acetate fraction. Silva-Silva et al. [40] when testing a fraction of *Arrabidaea chica* rich in flavonoids, also found that the treatment did not influence the increase of NO in macrophages infected with *L. amazonensis*. According to the authors, the flavonoids apigenin and luteolin could inhibit NO production by the suppressing of inducible nitric oxide synthase (iNOS) and cyclooxygenase-2 (COX-2). For Carneiro et al. [53], the role of NO in macrophage defense mechanisms can be specific for different species of *Leishmania*.

These defense mechanisms are complex and depend on the type of clinical manifestation, and disease progression [26]. Among the mechanisms implicated in the immune response of the host infected with *Leishmania* parasites, the expression of Th1 and Th2 cytokine profile stands out. The cytokine IL-12 is responsible for directing a Th1-type response, producing mainly IFN-γ, TNF-α, IL-12 and NO, activating macrophages, and consequently leading to the destruction of parasites. On the other hand, *Leishmania* is capable of directing the response to the Th2 profile, characterized by the production of IL-10 and IL-4 cytokines, which inhibit macrophages and lead to proliferation and resistance to infection [[54], [55], [56]].

In our findings, we verified the increase in TNF-α production by *Leishmania*-stimulated macrophages after treatment with *T. catappa* ethyl acetate fraction, which can be an important factor for the effectiveness of macrophages in reducing parasites. Conversely, we did not observe significant differences in the levels of IFN-γ, IL-1β and IL-12. Iheagwam et al. [21] verified that the aqueous extract of *T. catappa* leaves increased the levels of IL-6 and TNF-α in diabetic animals, suggesting an anti-inflammatory effect of the plant. However, Abiodun et al. [27] observed that the ethanolic extract of *T. catappa* inhibited the *in vitro* production of IL-1β and reduced the gene expression of pro-inflammatory mediators TNF-α, IL-23, IL-6 in *in vivo* assays*.* The increase in cytokines of Th1 profile, especially TNF-α, can be associated with the effective response of *T. catappa* in *Leishmania* infection *in vitro*, supporting the immunomodulatory effects of ethyl acetate fraction of *T. catappa*.

Phenolic compounds are main substances found in *T. catappa* ethyl acetate fraction and, the immunomodulatory effects these compounds are well evidenced in literature. Das et al. [57] observed encapsulated quercetin immunomodulatory effects on IFN-γ and IL-10 levels against *L. donovani.* Ellagic acid induced high immunomodulatory activity demonstrated by NO production, increased phagocytic capacity, lysosomal volume, and intracellular calcium [Ca^2+^] in macrophages infected with *L. major* [49]. Additionally, Keshav et al. [50] verified the action of ellagic acid against *L. donovani*. The compound was able to increase the levels of NO and reactive oxygen species (ROS), as well as increase the production of cytokines IL-12, IFN-γ and TNF-α. In addition, animals treated with ellagic acid had an expansion of CD4^+^ and CD8^+^ T cells and greater production of IgG2a antibodies. These results indicate that ellagic acid increased Th1-type immune response in *Leishmania*-infected animals. Therefore, the phytochemical compounds found in the ethyl acetate fraction of *T. catappa* may play an important role in immunomodulating macrophages stimulated with *L. amazonensis*.

During *Leishmania* infection, macrophages induce an oxidative burst producing mainly NO and ROS as defense mechanism, however these molecules at high levels can cause inflammation, resulting in tissue damage to host cells [58]. To combat oxidative stress, cells have developed antioxidant responses to counteract the damaging oxidative burst [58]. The Nrf2 is the transcription factor responsible for regulating genes in response to oxidative stress, especially HO-1 and ferritin [59,60]. HO-1 is an enzyme that catalyzes the degradation of heme emission, releasing iron and carbon monoxide, while ferritin is the main protein responsible for storing iron within the cell [61,62]. In leishmaniasis, iron acquisition is important for parasite survival and replication [[63], [64], [65]]. In this study, the *T. catappa* ethyl acetate fraction did not alter the expression of Nrf2, however, the expression of HO-1 and ferritin was downregulated in stimulated macrophages, suggesting a possible reduction in iron induced by *T. catappa* treatment that may interfere in *Leishmania* survival.

**Fig. 6:**
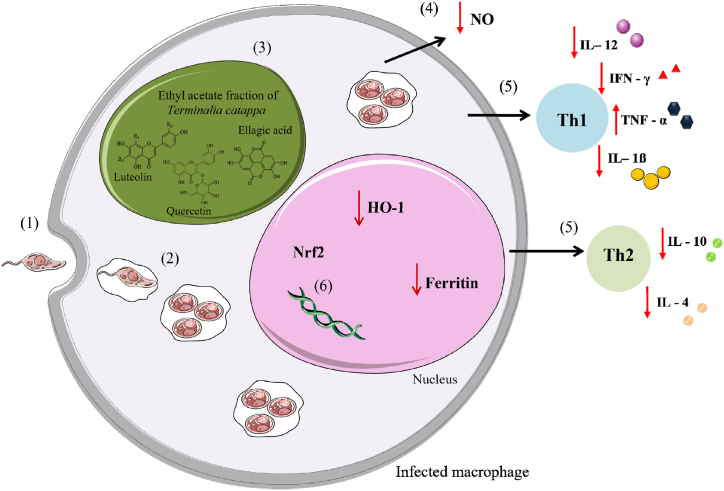
Immunomodulatory and pro-oxidant effects produced in macrophages stimulated with *Leishmania amazonensis* and treated with *Terminalia catappa* ethyl acetate fraction. Promastigotes are internalized by macrophages (1) and inside the cell, form the parasitophorous vacuole and transform into amastigotes (2). The ethyl acetate fraction inhibited parasite proliferation (3) but did not induce NO production (4). The treatment induced Th1 response, producing TNF-α, prevented the production of Th2 response (5), and decreased the expression of the HO-1 and ferritin genes in stimulated macrophages (6).

HO-1 expression activated by the Nrf2 pathway has been associated with survival and persistence of *Leishmania* infection [66,67]. Macrophages infected with *L. amazonensis* exhibited increased expression of Nrf2 and HO-1, which, together with higher levels of holotransferrin (holoTf), contributed to the persistence of infection [68]. Bichiou et al. [69] verified that macrophages infected with *L. major* also induced significant expression of Nrf2 and HO-1. The Nrf2, the main regulator of the antioxidant response, is strongly regulated in the macrophage-*Leishmania* interaction and the inactivation of this gene contributes to increased survival and multiplication of the parasite. Additionally, ferritin is the protein responsible for the intracellular storage of iron by *Leishmania*, therefore, preventing the absorption of iron by the parasite can be a strategy to control its proliferation. Based on that, our data suggest a pro-oxidant effect induced by the treatment with *T. catappa* that may contribute to the elimination of the intracellular forms. Fig. 6 illustrates the immunomodulatory and pro-oxidant mechanisms investigated in this study.

Studies have shown that plant extracts and phytochemical compounds target the Nrf2, HO-1 and ferritin genes potentially become effective in combating leishmaniasis. Tomiotto-Pellissier et al. [60] verified that *Caryocar coriaceum* extracts have a leishmanicidal effect against the promastigotes and amastigotes of *L. amazonensis* through the up-regulation of Nrf2, HO-1 and ferritin expression in infected macrophages, reducing the parasite replication rate. Rizk et al. [25] observed that amentoflavone (biflavonoid) did not alter Nrf2 expression, however, HO-1 was down-regulated and ferritin was up-regulated. It was expected that a decrease in HO-1 would be accompanied by a decrease in ferritin, since the release of iron results from HO-1 activity [70]. We observed that the ethyl acetate fraction of *T. catappa* decreased HO-1 expression and, consequently, ferritin. These findings demonstrate that HO-1 down-regulation is an important target for the development of new therapeutic strategies focusing on iron metabolism.

Given the importance of studies that seek to find new alternatives for treating leishmaniasis, mainly using medicinal plants, this work brought promising results. Additionally, this is the first report of antileishmanial activity of *T. catappa*. In this way, we conclude that *T. catappa* ethyl acetate fraction has leishmanicidal activity against *L. amazonensis*, acting on promastigote and intracellular amastigote forms. In addition, the treatment triggered immunomodulatory responses, mainly increased levels of TNF-α and reduced expression of antioxidant genes important for parasite survival within peritoneal macrophages, such as the ferritin and HO-1. Although our results have shown the leishmanicidal effect of the *T. catappa* ethyl acetate fraction from *in vitro* assays, *in vivo* studies are necessary to reinforce our findings.

Future developments in this line of research should be carried out in order to validate and implement the use of the extract and/or fractions obtained from *T. catappa* in the treatment of leishmaniasis, however, among the limitations, we highlight that as it is a model tested only *in vitro*, it should be analyzed in *in vivo* model.

## Funding

This work was supported by 10.13039/501100002322Coordination for the Improvement of Higher Education Personnel (Coordenação de Aperfeiçoamento de Pessoal de Nível Superior do Brazil - 10.13039/501100002322CAPES) [Finance Code 001], by the Fundação de Amparo à Pesquisa e Desenvolvimento Científico e Tecnológico do Maranhão - 10.13039/501100003758FAPEMA [grant number 02918/17- IECT], and by Fundação Carlos Chagas Filho de Amparo à Pesquisa do Estado do Rio de Janeiro [grant number 26/211.680/2021]. The APC (Fund for Conjoint Research Project) was funded by the Oswaldo Cruz Institute/10.13039/501100006507FIOCRUZ. Dr. Fernando Almeida-Souza is a postdoctoral research fellow and scholarship holder of FAPERJ [grant number E-26/203.513/2023]. Dra Katia da Silva Calabrese [grant number 315225/2021-1] and Dra Ana Lucia Abreu-Silva [grant number 313348/2019-9] are researcher productivity fellow of National Scientific and Technological Development Council (Conselho Nacional de Desenvolvimento Científico e Tecnológico – 10.13039/501100003593CNPq).

## Data availability statement

Data will be made available on request.

## CRediT authorship contribution statement

**Sandra Alves de Araújo:** Writing – review & editing, Writing – original draft, Visualization, Software, Methodology, Investigation, Conceptualization. **Carla Maria Pereira Silva:** Writing – review & editing, Methodology, Investigation. **Carolina Silva Costa:** Writing – review & editing, Methodology, Investigation. **Clarissa Sousa Costa Ferreira:** Writing – review & editing, Methodology, Investigation. **Helen Silva Ribeiro:** Writing – review & editing, Methodology, Investigation. **Aldilene da Silva Lima:** Writing – review & editing, Methodology, Investigation. **Cláudia Quintino da Rocha:** Writing – original draft, Visualization, Validation, Supervision, Software, Resources, Data curation, Conceptualization. **Kátia da Silva Calabrese:** Writing – review & editing, Visualization, Validation, Supervision, Software, Resources, Project administration, Funding acquisition, Data curation, Conceptualization. **Ana Lucia Abreu-Silva:** Writing – review & editing, Visualization, Validation, Supervision, Software, Resources, Project administration, Funding acquisition, Data curation, Conceptualization. **Fernando Almeida-Souza:** Writing – review & editing, Visualization, Validation, Supervision, Software, Project administration, Investigation, Data curation, Conceptualization.

## Declaration of competing interest

The authors declare that they have no known competing financial interests or personal relationships that could have appeared to influence the work reported in this paper.
