# Supplementary material for: Leishmanicidal and immunomodulatory activity of Terminalia catappa in Leishmania amazonensisin vitro infection
Source: Heliyon. 2024 Jan 12;10(2):e24622. doi: 10.1016/j.heliyon.2024.e24622 (PMC10835263; doi:10.1016/j.heliyon.2024.e24622)

***Supplementary material***

**S1.** Ellagic acid concentration analyzed by HPLC-UV and monitored at 254 nm.


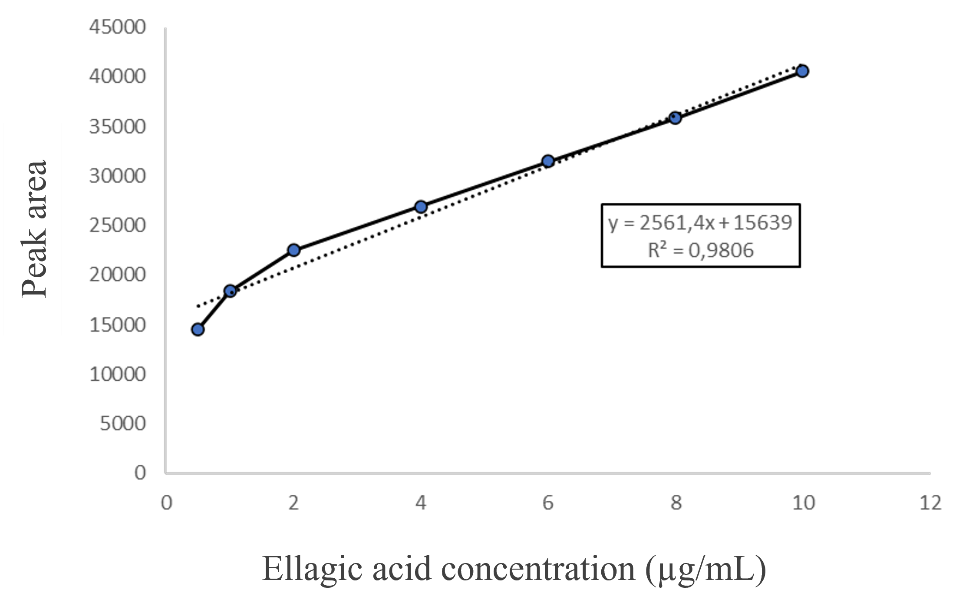


**S2.** Cytokines quantification in supernatant of cultured peritoneal macrophages treated with the ethyl acetate fraction of *Terminalia catappa* and stimulated by *L. amazonensis* or LPS for 72 hours.

|  | **Cytokine (pg/mL)** | | | | | |
| --- | --- | --- | --- | --- | --- | --- |
| **Group** | **TNF-α** | **IFN-γ** | **IL-1β** | **IL-12** | **IL-10** | **IL-4** |
| Non- stimulated and untreated | 35.00 ± 5.00 | 4.31 ± 2.39 | 54.16 ± 8.16 | 293.65 ± 222.10 | 92.85 ± 8.24 | 3.33 ± 0.72 |
| *L.a* and untreated | 13.75 ± 8.53 | 4.54 ± 0.64 | 129.16 ± 109.18 | 248.14 ± 167.15 | 67.85 ± 41.03 | 1.25 ± 0.00 |
| *L.a* + Ethyl acetate fraction (25 µg/mL) | 16.00 ± 6.51 | 4.99 ± 2.59 | 163.33 ± 75.15 | 528.88 ± 126.83 | 142.85 ± 120.79 | 4.00 ± 2.70 |
| *L.a* + Ethyl acetate fraction (50 µg/mL) | 20.00 ± 9.35 | 4.90 ± 1.88 | 140.00 ± 41.95 | 235.55 ± 134.34 | 128.57 ± 144.63 | 4.37 ± 3.14 |
| *L.a* + Ethyl acetate fraction (100 µg/mL) | 30.00 ± 6.12 | 3.81 ± 2.48 | 123.33 ± 108.01 | 558.33 ± 240.94 | 160.00 ± 120.79 | 6.25 ± 5.59 |
| LPS | 541.00 ± 145.05 | 35.00 ± 8.80 | 330.00 ± 93.29 | 196.29 ± 149.20 | 228.57 ± 123.30 | 2.91 ± 3.14 |

*L.a:* stimulated with *Leishmania amazonensis;* LPS: cells non-infected and stimulated with lipopolysaccharide

**S3.** Relative quantification of pro- and antioxidant genes in BALB/c peritoneal macrophages infected with *Leishmania amazonensis* and treated with *Terminalia catappa*. RT-qPCR analyses were performed to quantify the expression of Nfr2 mRNA.


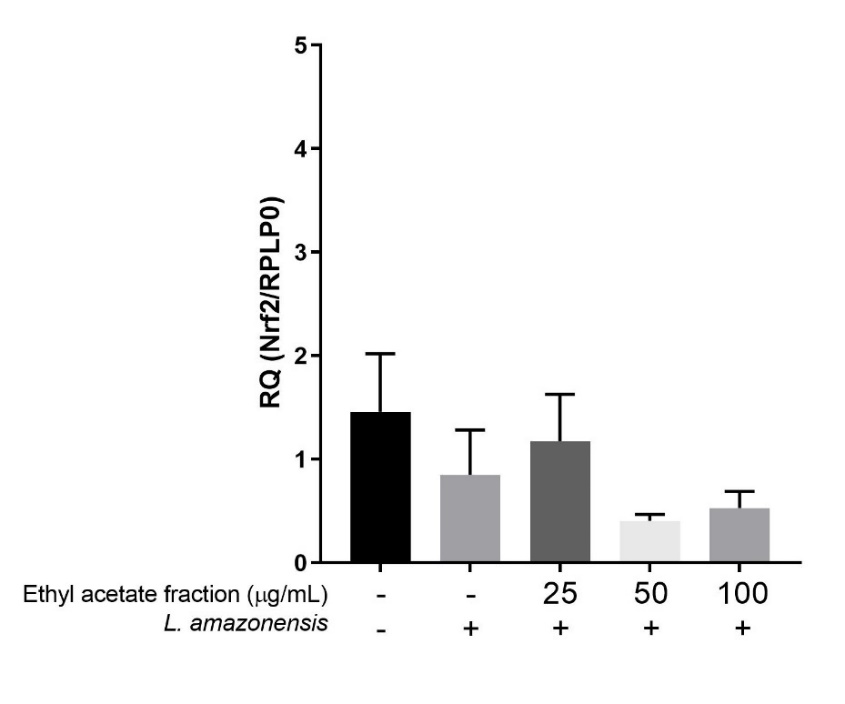

Supplement: Multimedia component 1 [file mmc1.docx]
